# Persistent excess mortality from lung cancer in patients with stage I non-small-cell lung cancer, disease-free after 5 years

**DOI:** 10.1038/sj.bjc.6600991

**Published:** 2003-05-27

**Authors:** F Pasini, G Verlato, E Durante, G de Manzoni, F Valduga, S Accordini, C Pedrazzani, A Terzi, G Pelosi

**Affiliations:** 1 Cattedra di Oncologia Medica, Università degli Studi di Verona, Italy; 2 Cattedra di Epidemiologia e Statistica Medica, Università degli Studi di Verona, Italy; 3 I Divisione Clinicizzata di Chirurgia, Università degli Studi di Verona, Italy; 4 Divisione di Chirurgia Toracica, Azienda Ospedaliera di Verona, Piazzale Stefani 1, 37126 Verona, Italy; 5 Divisione di Anatomia Patologica e di Medicina di Laboratorio, Istituto Europeo di Oncologia, Via Ripamonti, 435, 20141 Milano, Italy

**Keywords:** non-small-cell lung cancer, long-term survival, standardised incidence ratio, standardised mortality ratio

## Abstract

Among patients with non-small-cell lung cancer (NSCLC), those with pathological stage I have the best expectation of survival; however, survival is reduced to less than 50% in the long term. At present, it is unclear when patients can be reasonably defined as cured, and if they experience a higher incidence of malignant/nonmalignant diseases and a lower expectation of survival than the general population. A total of 134 stage I NSCLC patients, who had undergone resection at the Thoracic Surgery Unit of the General Hospital of Verona (north-eastern Italy) from October 1987 to December 1993, were still disease-free at 5 years. These subjects were further followed up, and morbidity and mortality rates were compared with those recorded in the general population of the same geographical area. The standardised incidence ratios (SIRs) for all malignancies and for lung cancer were higher than expected (2.39, 95% CI=1.6–3.5, *P*<0.001; 10.1, 95% CI=6.2–15.6, *P*<0.0001, respectively). The standardised mortality ratio (SMR) was also significantly increased (1.73, 95% CI=1.1–2.6, *P*=0.013). The excess mortality could be entirely explained by an increase in mortality from lung cancer (5.7, 95% CI=2.8–10.1, *P*<0.0001). This study shows that patients, resected for pathological stage I NSCLC and tumour-free after 5 years, have a higher incidence of new lung cancer compared with the general population, which in turn determines an excess in all-cause mortality in the following years.

Lung cancer is one of the leading causes of cancer death all over the world; as in most industrialised areas, also in the Veneto region (north-eastern Italy) the incidence accounts for about 75 cases per 100 000 inhabitants per year (Venetian Tumour Registry, 1999).

Among patients with non-small-cell lung cancer (NSCLC), those with pathological stage I have the best expectation of survival, since they do not present major negative prognostic factors such as incomplete resection, positive lymph nodes, distant metastases. At present, the standard therapy of stage I NSCLC is radical surgery. However, in the long term, the all-cause survival rate is reduced to less than 50%, due to deaths from relapses, new cancers and nonmalignant competing events.

It is unclear when patients can be reasonably defined as cured from their initial lung cancer; it is also unknown if they have a higher incidence of malignant and nonmalignant diseases or experience the same expectation of survival when compared to the general population.

To answer these questions, a series of patients with pathological stage I NSCLC, who had undergone resection in a single institution and were disease-free at 5 years, was further followed up, and morbidity and mortality rates were compared with the rates recorded in the general population of the same geographical area.

## PATIENTS AND METHODS

From October 1987 to December 1993, 246 consecutive patients underwent thoracotomy at the Thoracic Surgery Unit of the General Hospital of Verona, north-eastern Italy, for pathologically confirmed stage I (T1-2 N0 M0) NSCLC. None of the patients received neoadjuvant or adjuvant chemo/radiotherapy.

The hospital charts of all patients were reviewed; for those who were not routinely followed up, follow-up data were obtained by personal contact, or by a questionnaire sent to the family doctor, or relatives of a deceased patient. Information on disease occurrence, life status and cause of death, if any, was retrieved for all patients. Pathological slides were reviewed by a pathologist (PG) without knowledge of the clinical outcome.

This study focused on the outcome of 134 patients, out of the initial 246, who were disease-free at least 5 years after resection. These patients were followed up until 31 December 1999.

Disease and causes of death were coded according to the International Classification of Diseases, Ninth Revision (ICD9). The following health outcomes were considered: new diagnosis of cancer from any site (ICD9 code=140–208), new diagnosis of cancer of the trachea, bronchi and lung (ICD9 code=162), and death from any cause (ICD9 code=0–999).

Incidence and mortality rates in the cohort of 134 patients were compared with rates recorded in the Veneto region, the administrative area including Verona, through indirect standardisation (Rothman and Greenland, 1998).

Regional incidence rates were obtained from the Venetian Tumour Registry (1999) and regional mortality rates were obtained from the Italian Institute of Statistics (Italian Institute of Statistics, ISTAT, 2000).

### Statistical analysis

Standardised incidence ratios (SIRs) and standardised mortality ratios (SMRs) were computed by means of the program PYRS (person-years) version 1.3 (Coleman *et al*, 1986). Statistical significance of SMRs and SIRs was evaluated by the Poisson two-sided test (Verlato *et al*, 1999), and 95% confidence intervals (CI) were calculated by the exact method (Breslow and Day, 1987). Survival curves were estimated by the Kaplan–Meier method. All-cause survival time was defined as the time from surgery to death from any cause, disease-related survival time as the time from surgery to death from lung cancer, and disease-free survival time as the time from surgery to recurrence of the disease. When computing disease-related survival and disease-free survival, deaths from other causes were considered as censored observations. To allow graphic evaluation of the excess mortality, observed survival, estimated by the Kaplan–Meier method, was presented together with expected survival. The latter was computed by applying to the cohort under study age- and sex-specific mortality rates recorded in the general population of the area (Hakulinen, 1982).

## RESULTS

Data on complete survival, disease-related survival and disease-free survival times could be obtained for all 134 patients (18 females and 116 males). Median age at diagnosis was 62 years (range 35–80), and median follow-up was 96 months (range 61–144).

The 5- and 10-year disease-free survival percentages of the entire initial group (246 patients) were 62 and 49%, respectively; therefore, the 10-year cumulative risk of relapse was increased by 34% compared to that at 5 years. The 5- and 10-year disease-related survival percentages were 67 and 57%, respectively. All-cause survival percentages were 62 and 43%, respectively.

**Figure 1:**
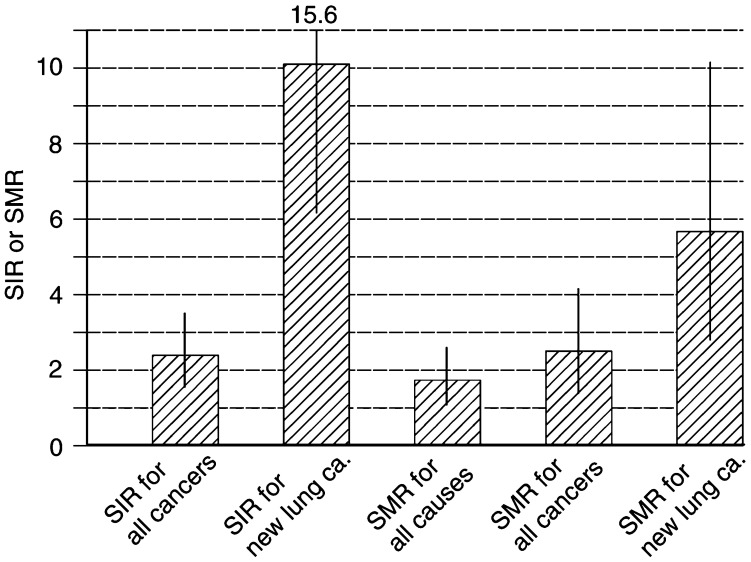
Standardised incidence ratios (SIRs) and standardised mortality ratios (SMRs) in the 134 patients who were alive and free from relapses of lung cancer 5 years after resection. Indirect standardisation was accomplished using as reference rates recorded in the Veneto region. Columns are values; bars are confidence intervals. ca.=cancer.

Beyond the first 5 years of follow-up, out of 134 patients 26 (19.4%) presented a second cancer, either lung cancer (*n*=17), other types of malignancies (*n*=6) or both (*n*=3), while the expected number was 10.87. The SIR (26/10.87=2.39, 95% CI=1.6–3.5) was significantly higher than expected (*P*<0.001) (Figure 1

).

Of these patients, 20 (14.9%) were diagnosed to have a new NSCLC appearing therefore after a 5-year tumour-free interval; this value was 10 times higher than that expected (SIR=20/1.98=10.1, 95% CI=6.2–15.6, *P*<0.0001).

Among the 134 patients, nine (6.7%) presented a second malignancy other than lung cancer. The SIR of extrapulmonary tumours after 5 years was comparable to that expected (9/9.7=0.93, 95% CI=0.4–1.8, *P*=1).

Overall, 23 patients died, while the expected number in the same series was 13.27. The SMR (23/13.27=1.73, 95% CI=1.1–2.6) was significantly increased (*P*=0.013); the excess mortality could be entirely explained by an increase in mortality due to all types of cancer (SMR=15/5.99=2.5, 95% CI=1.4–4.1, *P*=0.002). A more detailed analysis highlighted that the excess mortality was confined to deaths from lung cancer, which was nearly six times higher than expected (11/1.94=5.7, 95% CI=2.8–10.1, *P*<0.0001).

**Figure 2:**
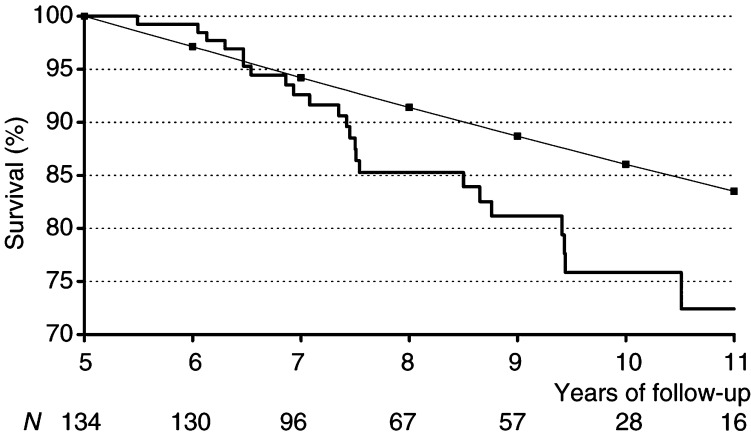
Kaplan–Meier estimates of survival probability (thick line) in a series of 134 patients who underwent surgery for lung cancer and who were apparently ‘cured’ after 5 years. Expected survival in the series according to mortality in the general population of the Verona area is also shown (thin line).

After 10 years of follow-up, the difference between the observed and the expected proportion surviving was about 0.1 (Figure 2

).

The two curves started to diverge after about 7 years of follow-up.

## DISCUSSION

This study evaluated the outcome of a series of consecutive patients with pathological stage I NSCLC who were free from their lung tumour 5 years after surgery.

In our series (Pasini *et al*, 2002), as well as in other reports, at 10 years the disease-related survival decreased by 10% compared to that at 5 years (from 67 to 57%) (Flehinger *et al*, 1992; Ichinose *et al*, 1993; Harpole *et al*, 1995; Martini *et al*, 1995). Most of the recurrences occurred in the first two or three years after diagnosis; since then, however, patients continued to relapse and die at a rate of 2–5%. In the period lasting from 5 to 10 years, the risk of relapse increased from 38 to 51%, a relative increment of 34%.

There is no general agreement on how these late events are to be classified (i.e. recurrences or second lung cancers). In our opinion, a disease-free interval of 5 years seems appropriate for the definition of second lung primaries, although the crucial point is the assessment of their incidence and impact on survival.

Moreover, NSCLC was not the unique cause of death because patients died also of competing events such as cardiovascular diseases, other non-neoplastic diseases and different types of tumours; eventually, the 10-year crude survival rate was only 43%.

Following this consideration, some questions may be raised in the subset of stage I NSCLC patients disease-free at 5 years. The first is whether this interval is sufficient to declare a patient cured; the second is whether these patients have a life expectancy equivalent to that of the general population.

To our knowledge, there are scanty data in the literature concerning the long-term outcome of such a cohort of patients; in particular, it is unclear whether the mentioned yearly recurrence rate (2–5%) is superior compared to the incidence of new lung cancers usually detected in the general population.

For this reason, we have compared the SIR for all types of cancers and the SMR of our study population with those of the general population of the Veneto region. This region is located in the north-east of Italy, an industrialised area in which lung cancer incidence is among the highest in Europe (about 75 cases per 100 000 inhabitants per year).

In the observed group, the SIR for all types of cancer was 2.4 times higher than that expected (*P*<0.001). The number of extrapulmonary tumours was similar to that of the control population (SIR=0.93), while the occurrence of second lung primaries in the study population was 10 times higher than in the controls (*P*<0.0001). Therefore, the excess of tumours was entirely due to second lung cancers.

This increased incidence of second lung primaries was not associated with confounding factors such as radiotherapy (i.e. in Hodgkin's disease) or chemotherapy, since none of these patients had received adjuvant treatment.

As for mortality, we found that the overall SMR was about 70% higher than that expected according to the mortality rates of the general population (*P*=0.013). Further analysis of the data elucidated the role of the various causes of death. It could be shown that second lung cancers caused in the study population an excess of mortality of about six times (*P*<0.0001), while mortality from other causes was similar. Moreover, as already reported, only a minority of the patients (about 30%) with second lung cancers survived beyond 3 years, mainly because of their advanced stage (Thomas and Rubinstein, 1993; Johnson, 1998).

The worse outcome can be seen in Figure 2, where the difference between observed and expected survival is about 0.1 after 10 years of follow-up.

In other words, most of the patients disease-free at 5 years can be considered cured; nevertheless, in a non-negligible proportion of them, second lung primaries showed an increased incidence with a low curability rate and were by far the leading cause of death.

These observations need validation with larger cooperative data, because, if confirmed, may provide new hints about the biological behaviour of the tumour.

They also emphasise the need for continued surveillance when planning the follow-up of patients in an attempt to detect new lesions at an early stage, when there are real chances of cure. On the other hand, the benefits of earlier intervention may represent a subset with differences in the biology of disease or other unknown factors (Thomas and Rubinstein, 1990; Faber, 1993; Antakli *et al*, 1995; Martini *et al*, 1999; Downey, 1999). Moreover, in the setting of prevention, other issues have to be considered, such as smoking cessation, carcinogenesis/chemoprevention studies and the use of new genetic tools able to characterise molecular markers of risk and drug activity. Integration of these strategies is necessary to reduce lung cancer mortality rate.

In conclusion, this study shows that patients with pathological stage I NSCLC tumour-free after 5 years, when compared with the general population, had a higher incidence of new lung cancers, which in turn explains the higher mortality observed in the following years.
